# Gene flow and genetic structure in the Galician population (NW Spain) according to *Alu *insertions

**DOI:** 10.1186/1471-2156-9-79

**Published:** 2008-12-02

**Authors:** Tito A Varela, José Fariña, Lois Pérez Diéguez, Rosa Lodeiro

**Affiliations:** 1 Laboratory of Anthropology, Faculty of Biology, University of Santiago de Compostela, 15782 Santiago de Compostela, Spain; 2 Laboratory of Anthropology, Faculty of Sciences, University of Vigo, Campus Lagoas, Marcosende, 36200 Vigo, Spain

## Abstract

**Background:**

The most recent *Alu *insertions reveal different degrees of polymorphism in human populations, and a series of characteristics that make them particularly suitable genetic markers for Human Biology studies. This has led these polymorphisms to be used to analyse the origin and phylogenetic relationships between contemporary human groups. This study analyses twelve *Alu *sequences in a sample of 216 individuals from the autochthonous population of Galicia (NW Spain), with the aim of studying their genetic structure and phylogenetic position with respect to the populations of Western and Central Europe and North Africa, research that is of special interest in revealing European population dynamics, given the peculiarities of the Galician population due to its geographical situation in western Europe, and its historical vicissitudes.

**Results:**

The insertion frequencies of eleven of the *Alu *elements analysed were within the variability range of European populations, while Yb8NBC125 proved to be the lowest so far recorded to date in Europe.

Taking the twelve polymorphisms into account, the GD value for the Galician population was 0.268. The comparative analyses carried out using the MDS, NJ and AMOVA methods reveal the existence of spatial heterogeneity, and identify three population groups that correspond to the geographic areas of Western-Central Europe, Eastern Mediterranean Europe and North Africa. Galicia is shown to be included in the Western-Central European cluster, together with other Spanish populations. When only considering populations from Mediterranean Europe, the Galician population revealed a degree of genetic flow similar to that of the majority of the populations from this geographic area.

**Conclusion:**

The results of this study reveal that the Galician population, despite its geographic situation in the western edge of the European continent, occupies an intermediate position in relation to other European populations in general, and Iberian populations in particular. This confirms the important role that migratory movements have had in the European gene pool, at least since Neolithic times. In turn, the MDS and NJ analyses place Galicia within the group comprised of Western-Central European populations, which is justified by the influence of Germanic peoples on the Galician population during the Middle Ages. However, it should also be noted that some of the markers analysed have a certain degree of differentiation, possibly due to the region's position as a 'cul-de-sac' in terms of Iberian population dynamics.

## Background

*Alu *sequences are a type of transposon from the SINEs group that are found in the genome of primates, into which they have inserted and expanded over the last 65 million years [1,2]. Some authors [1,3] maintain that only one subgroup of *Alu *elements (*Alu *master) are active in the generation of new copies, expanding in the human genome at a rate that has been estimated at one insertion for approximately every 20 births, a figure that supports the importance of *Alu *sequences in the structuring of the genome and their potential as mutagenic factors [4]. At present some 1,500,000 copies of these elements exist in the human species, representing approximately 11% of the total nuclear genome [2,5].

As a result of the evolution of the sequences inserted in the genome, at present various subfamilies of *Alu *elements are established that appear to have different genetic ages [6-8]. The most recent *Alu *insertions have a different degree of polymorphism in human populations [2,9], which makes them suitable genetic markers to be used in Human Biology studies. Furthermore, they are a type of polymorphism whose ancestral state is known, the alleles are identical by descent, as they are originated by a unique event, and they remain stable in phylogenetic terms, as well as being selectively neutral in the long term [10]. These characteristics have served to promote the use of the polymorphisms of *Alu *insertions as a means of analysing the origin and phylogenetic relations of modern human groups [11,12]. Interest in their use has increased in recent years due to their application in the identification of individual geographic origins [13-15].

**Figure 1:**
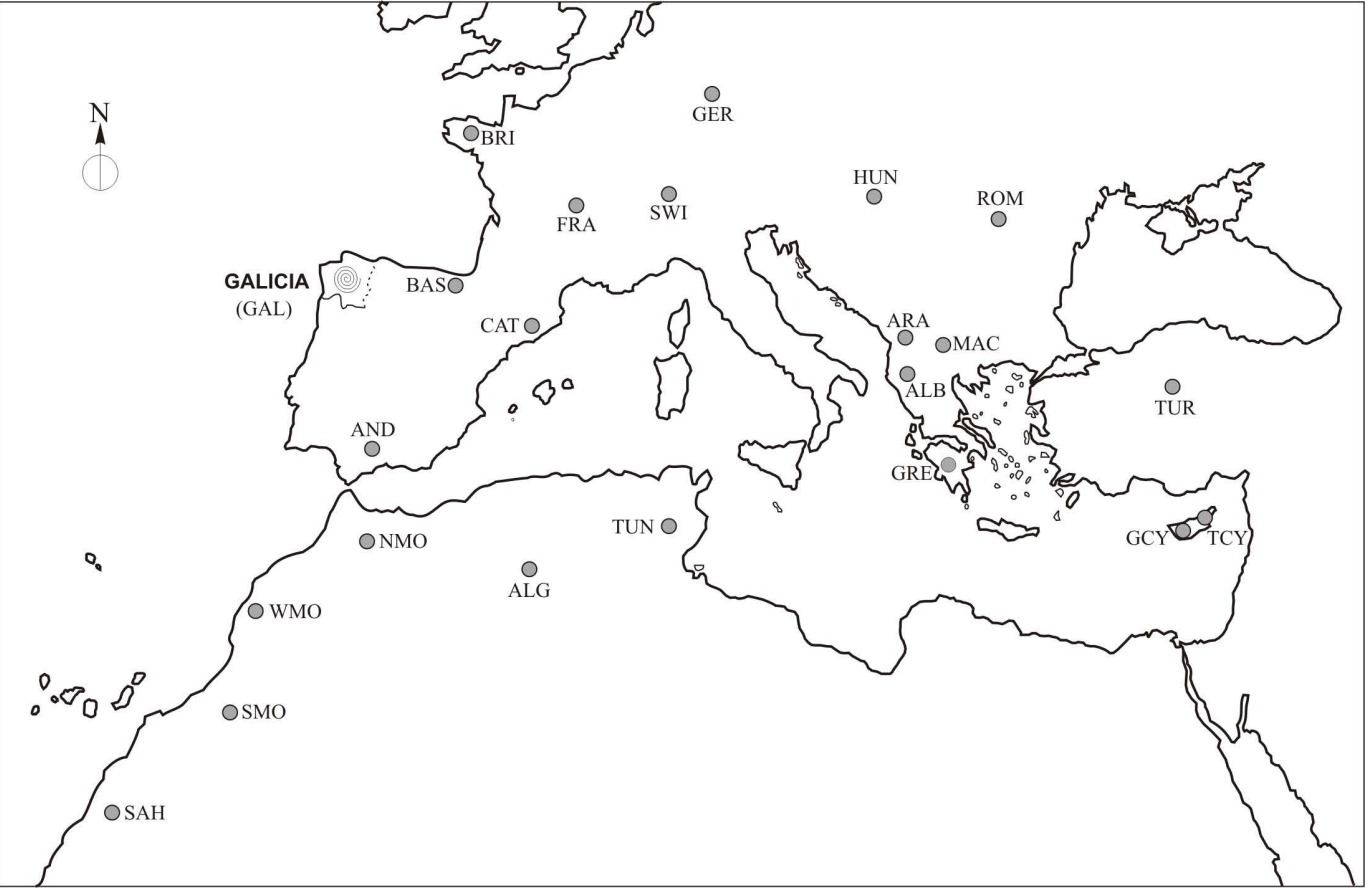
**Geographical location of Galicia (NW Spain) and the other 22 populations used in the comparative analyses.** ALB Albania, ALG Algeria, AND Andalusia, ARA Aromuns (Albania), BAS Basques (Spain), BRI Brittany, CAT Catalonia, FRA France, GAL Galicia, GER Germany, GRE Greece, GCY Greek Cypriots, HUN Hungary, MAC Macedonia, NMO North-Morocco ROM Romania, SAH Sahara, SMO Southeast-Morocco, SWI Swiss, TCY Turk Cypriots, TUN Tunisia, TUR Turkey, WMO West-Morocco.

Galicia is a historical region situated in the north-west Iberian Peninsula, and is the most westerly point of Europe (the Roman *Finis Terrae*) (Figure 1). Its particular geographic situation in a corner of the Iberian Peninsula, to some extent isolated from neighbouring territories, has been one of the factors that has conditioned the idiosyncrasy of the Galician population.

The first data available on the population of Galicia comes from stone tools, whose typology may be associated with a stage prior to modern forms of *Homo sapiens*, some 200,000–300,000 years BP, coinciding with the presence of similar tools in the north and centre of the Iberian Peninsula. Archaeological evidence corresponding to the Mesolithic and Neolithic periods is basically supported by megalithic dolmens found throughout the whole of the region [16], a megalithic culture that weakened at the end of the third millennium BC, coinciding with the creation of more permanent settlements and the beginning of trade with Brittany, the British Isles and the peoples of the Mediterranean. Iron metallurgy appears to have entered Galicia with the arrival of peoples from Central Europe, generically referred to as the Celts.

Roman domination of the north-western Iberian Peninsula, which began at the end of the first century BC, led to the territory becoming more structured, with a gradual disappearance of the hill fort settlements and a new political organisation in the most westerly Roman province of Hispania, the newly named *Gallaecia. *After the fall of the Western Roman Empire, in the early fifth century a series of Germanic peoples settled in the Iberian Peninsula, one of which, the Suebi, created a kingdom in *Gallaecia *which by the end of the sixth century was fully incorporated in Visigothic Spain, and would become configured as a feudal society based on agriculture and fishing.

At the beginning of the eighth century the peninsula was invaded by the Muslims, whose biological impact in Galicia and the rest of the Cantabrian cornice is presumed to be less than in other parts of the peninsula, as these northern regions were the refuge of the ruling Visigothic classes, who created the Kingdom of Asturias from where the 're-conquest' would begin. From the eighth to tenth century Galicia was repopulated, which served as a refuge for immigrants fleeing from the areas occupied by the Muslims. During this period the tomb of the Apostle St. James the Elder was discovered in Compostela, which led to the creation of an important pilgrimage route that connected Galicia with other European populations through the Way of St. James.

An important aspect in the dynamics of the Galician population in modern times was emigration, with large numbers of its inhabitants emigrating to Latin America from the middle of the nineteenth century, and to other European countries during the second half of the twentieth century. This migratory phenomenon affected the demographic structure of the region, and was one of the decisive factors leading to high levels of inbreeding, influencing the structure of consanguinity by increasing the number of marriages between closely related family members [17,18].

These historical vicissitudes and the region's geographic characteristics have led to the Galician population having a high degree of ethnicity, with its own language derived from Latin – *Gallego *– making it complex, unique, and of interest for anthropological research. The genetic structure of the Galician population has been analysed with respect to classic genetic and molecular polymorphisms [[19-25], amongst others] and nuclear and mitochondrial DNA [[26-29], amongst others], which have revealed the peculiarities of Galicia with respect to other Spanish populations.

The main objective of this study is to contribute towards a more thorough understanding of the genetic variability of the Galician population, characterising it through the study of twelve *Alu *polymorphisms, and thereby establishing its phylogenetic position with respect to other European and North African populations, offering new genetic data that contributes towards clarifying how the Iberian Peninsula was populated within the framework of population movements in the European areas in general, and the Mediterranean area in particular. The bibliography includes a work that has been published with the results of typing the *Alu *polymorphisms APOA1, HS2.43, HS4.65, Sb19.3 and Sb19.12 in the Galician population [30], carried out using a smaller sample than that used in this present study, and for which there is no evidence that the sample was taken using a criterion of population proportionality in all of the territory of Galicia; also, there are significant differences between the frequencies published and those given in this study, which in two cases (Sb19.3 and Sb19.12) are highly significant (*P *< 0.01). Another previously published work [31], which included the *Alu *polymorphisms APOA1, ACE and D1, was carried out with a different objective to that proposed in this present study, as it analysed Galicia's four administrative provinces independently, using small samples from each province, and did not make a characterisation of the Galician population as a whole.

## Results

The insertion frequencies for the twelve *Alu *elements studied in the Galician population are shown in Additional file 1, which also includes the genetic diversity values (GD) for each of these markers. In the Hardy-Weinberg equilibrium test, no significant deviations were found for any of the loci analysed. The *Alu *element whose frequency is closest to the fixation is Ya5NBC221 (0.961), with HS4.65 having the lowest frequency (0.014).

As would be expected, the lowest GD values in the genetic variability test correspond to the *Alu *insertions whose frequencies are closest to 0 and 1, as is the case with HS4.65 (GD = 0.028) and Ya5NBC221 (GD = 0.076), respectively. On the contrary, the highest values correspond to insertions whose frequencies are closest to 0.5, as is the case with Yb8NBC120 (Freq = 0.417; GD = 0.489) and ACE (Freq = 0.382; GD = 0.474). On quantifying the genetic diversity, taking the twelve *Alu *elements studied into account simultaneously, a GD value of 0.268 was obtained.

**Figure 2:**
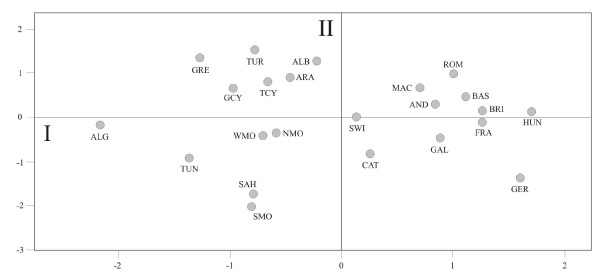
**Non-metric multidimensional scaling (MDS) applied to analyze genetic relationships among twenty-three populations.** The acronyms are the same as those used in Figure 1.

With the aim of representing the relative positions of the populations included in the analysis with respect to the genetic distances between them, calculated from the matrix of insertion frequencies, a non-metric multidimensional scaling (MDS) was carried out. The representation in the two-dimensional space configured by the first two vectors is shown in Figure 2. Axis I clearly separates the North African populations (North Morocco, West Morocco, Algeria, Tunisia, Southeast Morocco and the Sahara) and Eastern Mediterranean populations (the Aromuns of Albania, Albanians and Turks, Greek Cypriots, Turkish Cypriots and Greeks), which are situated in the negative area, while the remaining European populations (Switzerland, Catalonia, France, Germany and Hungary, Galicia, Rumania, Andalusia, Macedonia, Basque Country and Brittany) are situated in the positive area. In turn, axis II separates the first two groups, situating North African populations in the negative area, and all of those from the Eastern Mediterranean in the positive area.

**Figure 3:**
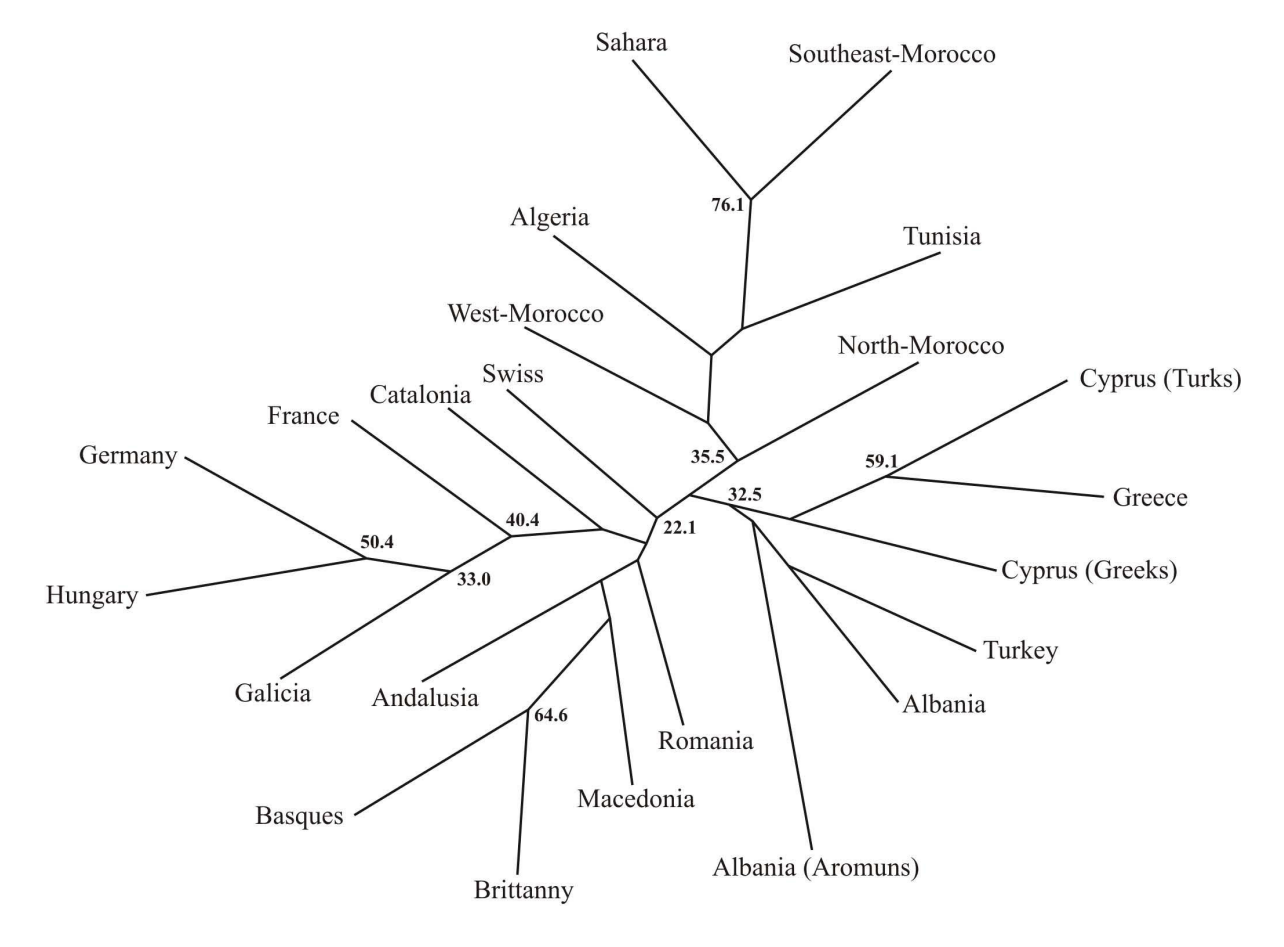
**Neighbour-joining tree of population relationships.** Numbers indicate (in percentage) the fraction of 10,000 bootstrap replicates that supported a particular grouping.

In order to verify if the relative positions of the populations in the space defined by the first two axes on the scalogram correspond to real groupings, a cluster analysis was carried out with Reynold's genetic distances matrix between pairs of populations, and a consensus tree was created (Figure 3), based on 10,000 trees generated by re-sampling, and based on the neighbour-joining method. The tree structure makes it possible to identify three nodes that cluster a further three population groups together, which in turn correspond to the three geographic areas differentiated in the MDS analysis.

Taking into account the fact that the previous results indicate that the populations included in the analyses may be differentiated into three groups, an analysis of molecular variance (AMOVA) was carried out, in order to evaluate the genetic structure of the populations with more precision, and the degree of subdivision or geographic distribution of the genetic diversity. The reliability of the three groups is revealed by the results of the AMOVA analyses, both in the case of the genetic between-group variation when the nine markers are considered simultaneously (χ^2^_18 _= 74.82; *P *< 0.01), and when each is considered separately (Additional file 2). In the latter case, the genetic variation among populations within groups (F_SC_) is not significant for any of the nine *Alu *insertions, while there are significant among-group differences (F_CT_) for six of them: ACE (1.46%, *P *< 0.01), APOA1 (2.02%, *P *< 0.01), B65 (1.88%, *P *< 0.01), D1 (1.32%, *P *< 0.01), HS3.23 (0.94%, *P *< 0.01), HS4.65 (4.67%, *P *< 0.01). The intra-population variance (F_ST_) was significant in six of the markers: A25 (98.98%, *P *< 0.05), ACE (98.61%, *P *< 0.05), APOA1 (97.44%, *P *< 0.01), B65 (97.80 *P *< 0.1) HS2.43 (99.12%, *P *< 0.05), and HS4.65 (95.79%, *P *< 0.01).

**Figure 4:**
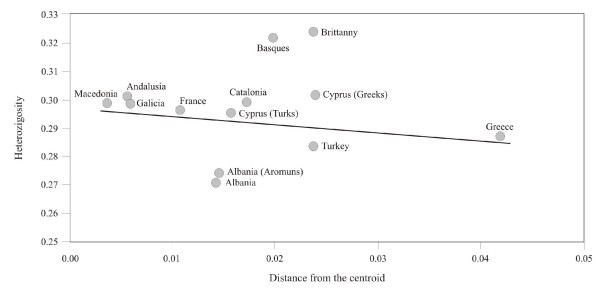
**Heterozygosity and distance from centroid in thirteen populations from the European Mediterranean area.** The line represents the expected regression according to the model of Harpending and Ward (1982).

The results obtained after applying the method of Harpending and Ward (1982) [32], shown in Figure 4, make it possible to deduce that the majority of the populations from the European Mediterranean area do not deviate to any major extent from the theoretical regression line, with Galicia at a very short distance from the centroid, while the Basque and Breton populations are the most distant from the theoretical prediction.

## Discussion

In general, reconstructions of the recent history of human populations based on genetic data, whether classic markers [33], nuclear DNA polymorphisms [34-37], or mitochondrial DNA [38], reveal a certain homogeneity in European populations, perhaps greater than in other continents. This conclusion is given further weight by lower differentiation index values amongst European populations, as is the case with the values of F_ST_, and the fact that on applying clustering methods to populations throughout the whole world, European populations are included in a more compact cluster that those from other continents, reflecting the small genetic distances between them.

With the aim of adding information to the proposed existence of a certain degree of uniformity amongst European populations, the Galician population was analysed, which occupies an extreme geographical position in Western Europe, and has been compared with other European and North African populations, as explained in the objectives of the study. *Alu *insertion polymorphisms were selected as they represent an important source of information about genetic diversity, both from the point of view of current diversity, and variability throughout the process of human evolution.

The frequency of the *Alu *insertions ACE, APOA1, D1, HS2.43, HS3.23 and HS4.65 in Galicia have intermediate values within the variability range of other populations in the Iberian Peninsula and the rest of Europe. As regards the *Alu *elements Sb19.3, Sb19.10, Sb19.12, Ya5NBC221, Yb8NBC120 and Yb8NBC125, less population information exists within their geographical surroundings, although what is known makes it possible to deduce that for the first five, the Galician population has values that are slightly above the average. The exception is Yb8NBC125, for which the lowest values known to date in Europe were recorded in Galicia. These results may be interpreted as evidence that Galicia shares the genetic homogeneity of Iberian and European populations, and it is most likely that when the number of populations typed for the *Alu *insertion Yb8NBC125 increases, the Galician population will be included within the European variability range, as is the case with the other *Alu *elements. Nevertheless, it is important to note that as a result of its geographic position, the Galician population represents a 'cul-de-sac' in terms of the historical dynamics of populations in the peninsula, which justifies a certain degree of difference with respect to other genetic markers [[24-27], amongst others].

In the first level of comparison, using the MDS and NJ analyses, the presence of three clusters was observed, which are coherent with the genetic diversity that would be expected as a result of historical dynamics and the exchange of populations. According to the hypothesis of demic expansion which followed the development of agriculture, originating in the Middle East and which then affected Central and Mediterranean Europe from the beginning of the Neolithic Period, the genetic diversity of populations from the Late Palaeolithic, probably resulting from drift, underwent a slow but continuous process of homogenisation as a result of the massive arrival of immigrants [39]. The exchanges of population that occurred during the Roman Empire and later periods also had similar effects, although these had a greater impact on the gene pool in Western Europe, affecting Eastern Europe to a lesser degree. Despite the fact that Neolithic expansion had the same effect in Northern Africa as in Europe, the Straits of Gibraltar acted as a barrier between the two continents, limiting gene flow between North-western Africa and Western Europe through the Iberian Peninsula [40-42]. This justifies the fact that North African populations appear in the MDS and NJ analyses as a separate group from European populations, although their position with respect to dimension I of the scalogram indicates a greater difference with respect to Western-Central Europe.

With regard to the Galician population, the MDS and NJ analyses confirm its position as clustered with other Spanish populations and those of Western-Central Europe. This result may be explained by common historical dynamics, mainly based on two events that occurred during the Middle Ages, with the invasion of Germanic peoples, especially the Suebi and Visigoths, and the beginning of pilgrimages along the Way of St. James, an important route for cultural exchange, and even the appearance of permanent settlements of Frankish peoples [43]. As would be expected, these types of relationships would have been accompanied by biological exchanges that would have brought the gene pool of the receptor population closer to that of the immigrant population.

The results of the AMOVA analysis support the reliability of the three clusters defined by the MDS and NJ, given the statistical significance of the between-group variation (F_CT_) for six of the nine markers, while the between-group differences (F_SC_) are not significant for any of them.

The method of Harpending and Ward (1982) [32], which uses the theoretic regression of population heterozygosity and distance to the centroid as a valid indicator to analyse the geographic model of genetic structure in populations, has been applied to the populations of Mediterranean Europe in order to evaluate the relationship of Galicia with other populations in this area. In this case, the Galician population is very close to the theoretical prediction line, as is the case with most of the populations in its geographical area included in the analysis, even Greece and Turkey, two of the populations situated to the eastern extreme of the area. The samples from Albania and Albanian Aromun are low outliers, which is interpreted as being indicative of both populations being receivers of a limited gene flow from the area; the samples from Basques and Bretons are high outliers, a position that does not necessarily imply that they recently received a greater external contribution, but which is indicative of a series of genetic differences with respect to the populations included in the analysis that may be due to the effect of the genetic contributions inherent to their origin.

## Conclusion

Based on the study of the twelve *Alu *polymorphisms included in this work, it may be deduced that the Galician population occupies an intermediate position in relation to Iberian and European populations, although considering its peculiar situation in the extreme west of Europe and its condition as a 'cul-de-sac' within the Iberian Peninsula, it has extreme values for some markers. The predominance in Galicia of intermediate values in the frequencies of *Alu *insertions contrasts with its geographic situation in the extreme west of the Iberian Peninsula, a fact which, in the light of current knowledge, should be interpreted as a consequence of the interaction of autochthonous and migratory phenomena. This fact supports the important role that migratory movements have played in European populations, at least since the Neolithic period. The MDS and NJ analyses situate Galicia within the group formed by western European populations, revealing the influence that invasions by Germanic peoples may have had on the Galician population in the Early Middle Ages, and to a lesser extent, European pilgrimages to Santiago de Compostela during the Late Middle Ages.

## Methods

### Population sample

Galicia borders to the south with Portugal, to the east with the regions of Asturias and León, while the north and west face onto the Atlantic Ocean. Today the region is divided into four administrative provinces: A Coruña, Lugo, Ourense and Pontevedra. It covers a total of 29,575 Km^2 ^and its population, according to the last census in January 2008, is 2,783,100, equivalent to a density of 94.10 Inhabitants/km^2^. Two of the peculiarities of Galicia's demographics are the eminently rural nature of its population and the highly scattered nature of its settlements; a particularly significant feature is that in the last third of the twentieth century, it had more than 31,000 centres of population, with two thirds of the population living in settlements of less than 1000 inhabitants [44,45], meaning that statistically there is more than one centre of population per Km^2^.

**Figure 5:**
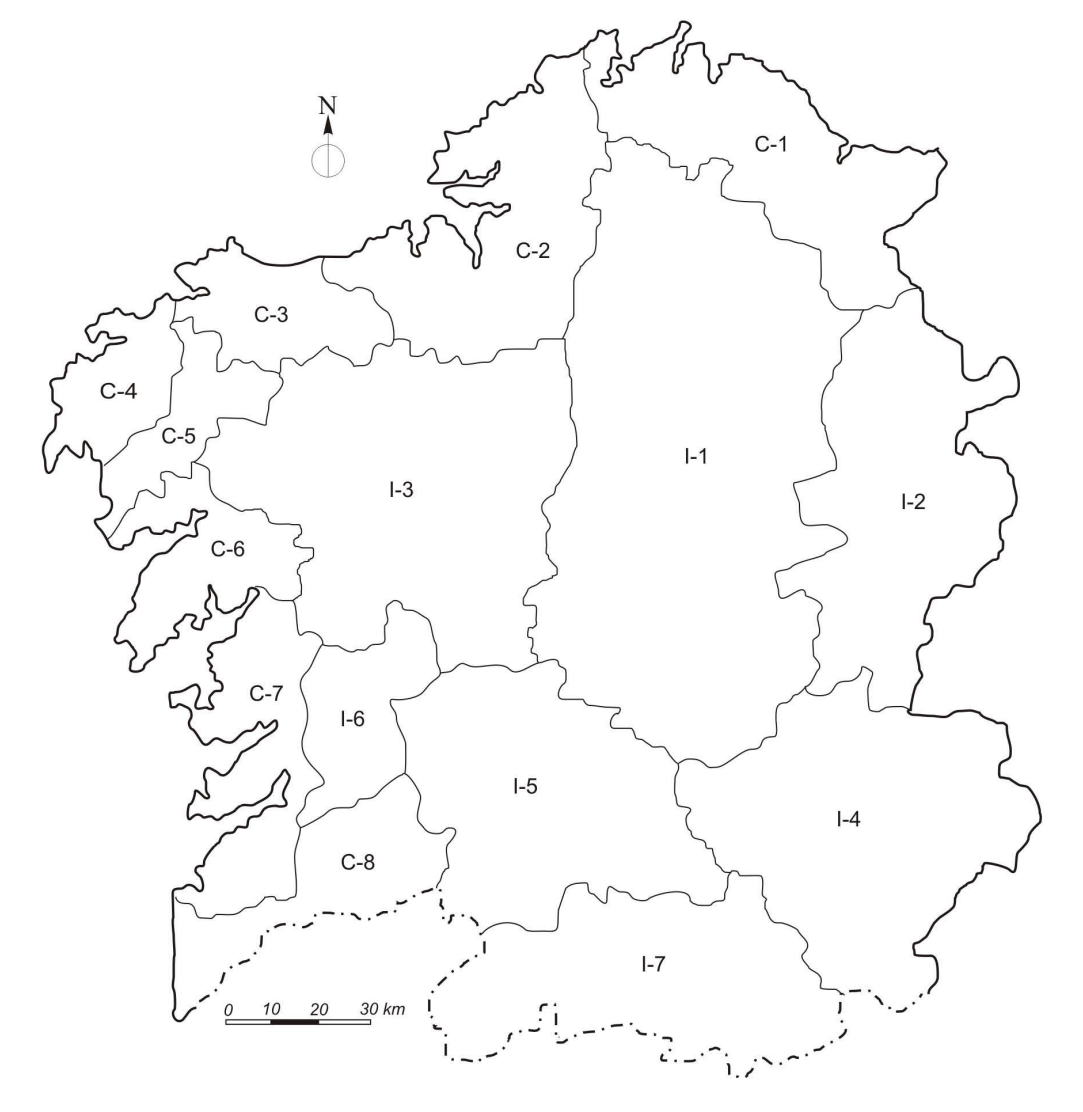
Location within Galicia of the fifteen natural districts into which the territory was divided: C-1 A Mariña, C-2 Golfo Ártabro, C-3 Bergantiños, C-4 Fisterra, C-5 Xallas, C-6 Santiago Oeste, C-7 Rias Baixas, C-8 Baixo Miño, I-1 Galicia Central, I-2 Serras Orientais, I-3 Santiago Este, I-4 Serras Surorientais, I-5 Ourense, I-6 Terra de Montes, I-7 A Limia/Verín.

In an effort to guarantee that the sample analysed represented the variability of the Galician population, the territory was divided according to a geographic criterion into eight coastal districts, and seven interior districts (Figure 5) [46]. The total number of donors in the fifteen districts was 216 (n = 432 chromosomes) distributed in such a way that the number of donors in each, with respect to the total number included in the sample, had the same proportionality ratio as the number of inhabitants in the district with respect to the total population of Galicia (Additional file 3).

Also, the conditions of anonymity and confidentiality established by the Ethics Committee of the University of Santiago de Compostela were strictly adhered to during the process of obtaining the samples.

### DNA typing

The genetic characterisation of the Galician population was carried out by analysing the polymorphism of twelve autosomal *Alu *insertions (ACE, APOA1, D1, HS3.23, HS2.43, HS4.65, Sb19.3, Sb19.10, Sb19.12, Ya5NBC221, Yb8NBC120 and Yb8NBC125), typed using DNA isolated from peripheral venous blood extracted from voluntary healthy donors, without close biological parentage and with a level of Galician autochthony up to the third preceding generation.

The extraction of genomic DNA from the leukocyte fraction of the blood samples was carried out using the GFX Genomic Blood DNA Purification kit from GE Healthcare. The extracted DNA was stored at -20°C.

The twelve *Alu *insertions were typed by PCR amplification, using the protocols and primers previously established by other authors, then carrying out electrophoresis in 2% agar gel and staining using ethidium bromide (0.5 μl/ml); visualisation and documentation of the gels was carried out using the Gel Doc 2000 system (BioRad) for gel analysis and documentation.

### Analysis of genetic data

The allele frequencies of the twelve *Alu *loci typed were calculated by direct counting. The Hardy-Weinberg equilibrium test was carried out using the exact test to calculate the *P *values from the Markov chains in the Monte Carlo method [47] using version 3.11 (2006) of the Arlequin programme from Excoffier et al. (2005) [48]. Intrapopulation variability was estimated using the unbiased index of genetic diversity (GD), carrying out the calculation using the method described by Weir (1996) [49].

In order to analyse the phylogenetic relationships of the Galician population with other populations in its geographic surroundings, a comparative analysis at two levels was carried out, using data on *Alu *insertion frequencies in populations from the selected areas that had been previously published in the scientific literature.

The first level was carried out including populations from Central and Southern Europe, as well as Northern Africa, together with the Galician population in order to make the comparison (Figure 1). It should be noted that no populations were found in which the twelve *Alu *insertions analysed in this study were typed, meaning that in the comparative analysis we omitted the *Alu *polymorphisms Sb19.3, Sb19.10, Sb19.12, Ya5NBC221, Yb8NBC120 and Yb8NBC125, and in order to use a number of markers that provides more reliable results, the three *Alu *polymorphisms were included (TPA25, A25 y B65) that had been previously studied in Galicia by this team [50]. This meant it was possible to carry out the analyses using the frequencies of nine markers (TPA25, A25, B65, ACE, APOA1, D1, HS3.23, HS2.43, HS4.65) in 22 populations [11,41,51-53] from the geographic area mentioned.

In the second level, using the same markers, a comparison was made exclusively between the Galician population and European Mediterranean populations, with the aim of analysing the model of the genetic structure in the populations in this geographic area.

The relationships of affinity between the Galician population and other European and North African populations were analysed using non-metric multidimensional scaling (MDS) (Kruskal 1964) [54] based on the inter-population Euclidean distances calculated from the matrix of allele frequencies. In this case the ALSCAL programme included in the SPSS v 16.0 package was used.

Reynold's genetic distance matrix between pairs of populations [55] formed the initial basis for population clustering using the neighbour-joining method (NJ) of Saitou and Nei (1987) [56], whose clustering reliability was established using a bootstrapping with 10,000 iterations; Reynold's distance calculation method, the NJ clustering algorithm and the bootstrap are all included in the Phylip 3.6 phylogenetic analysis package [57]. The cluster analysis makes it possible to verify if the relative positions of the populations shown on the scalogram correspond to specific population clusters.

In order to determine if the genetic variation is structured spatially, in the first comparison the MDS and NJ analyses were complemented with an analysis of molecular variance (AMOVA) from Excoffier et al. (1992) [58]. The AMOVAs for each *Alu *element were carried out using the Arlequin 3.11 programme [48]. With the aim of evaluating the global significance of the three components of the variance (F_CT_, F_SC _and F_ST_), a simultaneous contrast was carried out, taking into account the nine genetic markers included in the analysis, whereby for each component the critical global level (Pn) was calculated based on the critical levels (p_i_) obtained for each of the nine *Alu *elements (n). The statistic used is defined by the formula:

$$P_{n}=\sum_{i=1}^{n}\left(-2\ln p_{i}\right)$$

whose distribution fits a chi-square distribution with 2n degrees of freedom.

In order to study the geographic model of the genetic structure of the populations from the European Mediterranean area, in the second level of analysis the model of Harpending and Ward (1982) [32] was used, which graphically represents the expected frequency of heterozygotes, according to the Hardy-Weinberg law, for each population with respect to the distance to the centroid, *r*_*i*_, which is given by the formula

*r*_*i *_= (*p*_*i *_- *P*)^2^/[*P*(1-*P*)]

where *p*_*i *_and *P*, respectively, are the frequency of the *Alu *insertion in the population *i*, and in all of the populations included in the analysis as a whole.

In turn, Harpending and Ward propose an island model of population structure in which there is a lineal relationship between heterozygosity and the distance to the centroid:

*h*_*i *_= *H*(1-*r*_*i*_)

where *h*_*i *_and *H *correspond to the heterozygosity value in the population *i *and in all of the populations as a whole.

## Authors' contributions

All of the authors responsible for producing this work have made an equal contribution, and they have agreed on the final manuscript.

## Supplementary Material

Additional file 1 **Table 1.** This file contains the data on insertion frequencies and genetic diversity of the Galician population. Click here for file

Additional file 2 **Table 2.** Results of the AMOVA analysis in three population groups based on a geographical criterion and according to nine *Alu *markers Click here for file

Additional file 3 **Table 3.** Number of inhabitants in the districts of Galicia and the contribution made by each of them to the population sample Click here for file
