# Supplementary material for: Gene flow and genetic structure in the Galician population (NW Spain) according to Alu insertions
Source: BMC Genet. 2008 Dec 2;9:79. doi: 10.1186/1471-2156-9-79 (PMC2630999; doi:10.1186/1471-2156-9-79)
Supplement: Additional file 1 — Table 1. This file contains the data on insertion frequencies and genetic diversity of the Galician population. [file 1471-2156-9-79-S1.doc]

| *Alu* locus |  | Frequency ± SE |  | GD |
| --- | --- | --- | --- | --- |
| ACE |  | 0.382 ± 0.023 |  | 0.474 |
| APOA1 |  | 0.956 ± 0.010 |  | 0.085 |
| D1 |  | 0.354 ± 0.023 |  | 0.460 |
| HS2.43 |  | 0.069 ± 0.012 |  | 0.130 |
| HS3.23 |  | 0.861 ± 0.017 |  | 0.240 |
| HS4.65 |  | 0.014 ± 0.006 |  | 0.028 |
| Sb19.3 |  | 0.928 ± 0.012 |  | 0.134 |
| Sb19.10 |  | 0.354 ± 0.023 |  | 0.460 |
| Sb19.12 |  | 0.331 ± 0.023 |  | 0.445 |
| Ya5NBC221 |  | 0.961 ± 0.010 |  | 0.076 |
| Yb8NBC120 |  | 0.417 ± 0.024 |  | 0.489 |
| Yb8NBC125 |  | 0.109 ± 0.015 |  | 0.195 |
| GD value for all loci = 0.268 | | | | |
