# Supplementary material for: Gene flow and genetic structure in the Galician population (NW Spain) according to Alu insertions
Source: BMC Genet. 2008 Dec 2;9:79. doi: 10.1186/1471-2156-9-79 (PMC2630999; doi:10.1186/1471-2156-9-79)
Supplement: Additional file 2 — Table 2. Results of the AMOVA analysis in three population groups based on a geographical criterion and according to nine Alu markers [file 1471-2156-9-79-S2.doc]

|  |  |  |  |  |  |  |
| --- | --- | --- | --- | --- | --- | --- |
| *Alu* locus |  | *F_CT_* ^a^(%) |  | *F_SC_* ^b^(%) |  | *F_ST_* ^c^(%) |
| A25 |  | 0.39 NS |  | 0.64 NS |  | 98.98* |
| ACE |  | 1.46** |  | -0.08 NS |  | 98.61* |
| APOA1 |  | 2.02** |  | 0.55 NS |  | 97.44** |
| B65 |  | 1.88** |  | 0.32 NS |  | 97.80** |
| D1 |  | 1.32** |  | -0.38 NS |  | 99.06 NS |
| HS2.43 |  | 0.31 NS |  | 0.58 NS |  | 99.12* |
| HS3.23 |  | 0.94** |  | -0.30 NS |  | 99.36 NS |
| HS4.65 |  | 4.67** |  | -0.46 NS |  | 95.79** |
| TPA25 |  | 0.02 NS |  | -0.33 NS |  | 100.31 NS |
| Overall critical values |  | 74.82** |  | 20.85 NS |  | 52.75** |
|  |  |  |  |  |  |  |
| * Statistical significance at *P* < 0.05; ** Statistical significance at *P*< 0.01; NS, nonsignificant  ^a^ *F_CT_*Genetic variation among groups  ^b^ *F_SC_*  Genetic variation among populations within groups  ^c^ *F_ST_*  Genetic variation among individuals within populations  ^1^ Population clusters: Western-Central European (Andalusia, Britanny, Catalonia, France, Galicia, Germany, Hungary, Iberian Basques, Macedonia, Romania, Swiss), Eastern European Mediterranean (Albania, Aromun Albanians, Greece, Greek Cypriot, Turkey, Turk Cypriot); North Africa (Algeria, North-, Southeast- and West- Morocco, Sahara, Tunisia) | | | | | | |
