# Supplementary material for: Gene flow and genetic structure in the Galician population (NW Spain) according to Alu insertions
Source: BMC Genet. 2008 Dec 2;9:79. doi: 10.1186/1471-2156-9-79 (PMC2630999; doi:10.1186/1471-2156-9-79)
Supplement: Additional file 3 — Table 3. Number of inhabitants in the districts of Galicia and the contribution made by each of them to the population sample [file 1471-2156-9-79-S3.doc]

|  |  |  |  |  |  |  |
| --- | --- | --- | --- | --- | --- | --- |
| Code |  | District |  | Inhabitants |  | Number  of donors |
| C-1 |  | A Mariña |  | 90,598 |  | 7 |
| C-2 |  | Golfo Ártabro |  | 598,310 |  | 47 |
| C-3 |  | Bergantiños |  | 72,094 |  | 6 |
| C-4 |  | Fisterra |  | 42,058 |  | 3 |
| C-5 |  | Xallas |  | 26,333 |  | 2 |
| C-6 |  | Santiago Oeste |  | 139,607 |  | 11 |
| C-7 |  | Rías Baixas |  | 722,568 |  | 56 |
| C-8 |  | Baixo Miño |  | 115,712 |  | 9 |
|  |  |  |  |  |  |  |
| I-1 |  | Galicia Central |  | 262,482 |  | 20 |
| I-2 |  | Serras Orientais |  | 25,510 |  | 2 |
| I-3 |  | Santiago Este |  | 306,991 |  | 24 |
| I-4 |  | Serras Surorientais |  | 50,302 |  | 4 |
| I-5 |  | Ourense |  | 230,301 |  | 18 |
| I-6 |  | Terra de Montes |  | 28,187 |  | 2 |
| I-7 |  | Verín-A Limia |  | 61,480 |  | 5 |
|  |  | Total of Galicia |  | 2,772,533 |  | 216 |
|  |  |  |  |  |  |  |
